# Bacteria in cancer therapy: A new generation of weapons

**DOI:** 10.1002/cam4.4799

**Published:** 2022-05-06

**Authors:** Jun Ying Fan, Yuan Huang, Yi Li, Tobias Achu Muluh, Shao Zhi Fu, Jing Bo Wu

**Affiliations:** ^1^ Department of Oncology The Affiliated Hospital of Southwest Medical University Luzhou Sichuan P.R. China; ^2^ Department of Nuclear Medicine The Affiliated Hospital of Southwest Medical University Luzhou Sichuan P.R. China; ^3^ Academician (Expert) Workstation of Sichuan Province Luzhou Sichuan P.R. China

**Keywords:** bacteria‐mediated cancer therapy, cancer therapy, immunity, tumor

## Abstract

Tumors are presently a major threat to human life and health. Malignant tumors are conventionally treated through radiotherapy and chemotherapy. However, traditional therapies yield unsatisfactory results due to high toxicity to the normal cells, inability to treat deep tumor tissues, and the possibility of inducing drug resistance in the tumor cells. This has caused immunotherapy to emerge as an effective and alternate treatment strategy. To overcome the limitations of the conventional treatments as well as to avert the risk of various drug resistance and cytotoxicity, bacterial anti‐tumor immunotherapy has raised the interest of researchers. This therapeutic strategy employs bacteria to specifically target and colonize the tumor tissues with preferential accumulation and proliferation. Such bacterial accumulation initiates a series of anti‐tumor immune responses, effectively eliminating the tumor cells. This immunotherapy can use the bacteria alone or concomitantly with the other methods. For example, the bacteria can deliver the anti‐cancer effect mediators by regulating the expression of the bacterial genes or by synthesizing the bioengineered bacterial complexes. This review will discuss the mechanism of utilizing bacteria in treating tumors, especially in terms of immune mechanisms. This could help in better integrating the bacterial method with other treatment options, thereby, providing a more effective, reliable, and unique treatment therapy for tumors.

## Graphical abstract

Cancer cell progression and Bacteria therapy within the tumor microenvironment. The formation of cancer cells resulting from uncontrolled growth of normal cells. Bacteria and cancer cells within the surrounding microenvironment interaction lead to various changes in tumor‐infiltrating immune cells which promote tumor regression.

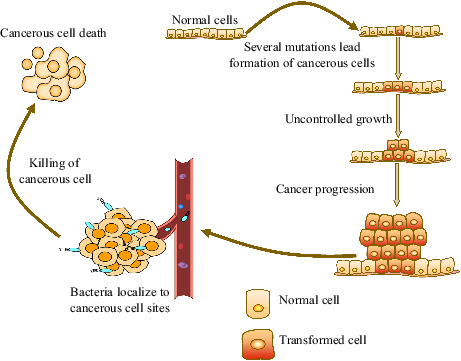

## 1 INTRODUCTION

The presently available treatment for tumors is not only toxic to the normal cells and fails to penetrate to the deep layers of the tumor tissues, but it also induces drug resistance in the tumor cells. This necessitates exploring alternate as well as effective treatments with low side effects. William Bradley Coley, the father of immunotherapy, observed the remission of malignancy after erysipelas infection in 1891. He devised a new method of treating cancer patients by intratumoral injection of live *Streptococcus pyogenesis*, thereby founding a novel era of cancer immunotherapy.^1^ The immunotherapy technique was further improvised by targeting the specific tumor cells using bacteria, which was termed bacterial immunotherapy.^2^ Despite the benefits, there has been a dearth of comprehensive understanding of the immune mechanism as well as a severe risk of infection by pathogenic bacteria when using bacterial immunotherapy. This has replaced bacterial immunotherapy with radiotherapy and chemotherapy.^3^, ^4^ With the advent of immune checkpoint and chimeric antigen receptor T‐cell therapies, immunotherapy has resulted in the emergence of a turning point in cancer immunotherapy.^5^ Although bacteria‐mediated immunotherapy has not been widely used in clinical practice, recent studies have demonstrated bacteria to possess an enormous potential to combat different cancers. In recent years, several animal experiments have proven bacterial therapy to effectively induce tumor regression and cure them. Bacterial therapy can stimulate the immune system and amplify the immune effect, thereby clearing the distant tumor tissues and preventing the recurrence of cancer.^6^ Several facultative or obligate anaerobic bacteria, like *Salmonella*, *Bifidobacterium*, *Clostridium*, *Listeria*, and *Escherichia coli*, can inherently target tumors and induce pathogenicity. The Bacillus Calmette–Guèrin (BCG) vaccine is currently the only clinically used bacteria‐mediated immunotherapy. This vaccine is delivered directly to the bladder for conventionally treating non‐invasive bladder cancer. Several studies suggest that BCG therapy can lessen the recurrence and progression of non‐muscle‐invasive bladder cancer.^7^ Tumor metastasis mainly accounts for the death of the patients. Bacterial‐mediated cancer therapy affects metastatic cancer, multidrug resistance in cancer, and cancer‐immune evasion.^6^

Bacteria‐mediated cancer therapy is indeed a promising treatment strategy for overcoming many of these current limitations evident with conventional therapy. However, the mechanisms of bacterial‐mediated cancer therapies are extremely complicated. This review focuses on the possible mechanisms of bacteria‐mediated cancer therapy, particularly highlighting the immune mechanisms. It aims to enhance our understanding of this treatment strategy and provides a special immunotherapy strategy for tumors.

## 2 BACTERIA TARGET THE TUMOR MICROENVIRONMENT

Most solid tumors are subjected to hypoxia and necrosis owing to the following two points: ^1^ The rate of growth of the tumor cells outgrows the rate of angiogenesis,^2^ Tumor angiogenesis is poor and heterogeneous, leading to the insufficient supply of oxygen to the tumor.^8^

**FIGURE 1:**
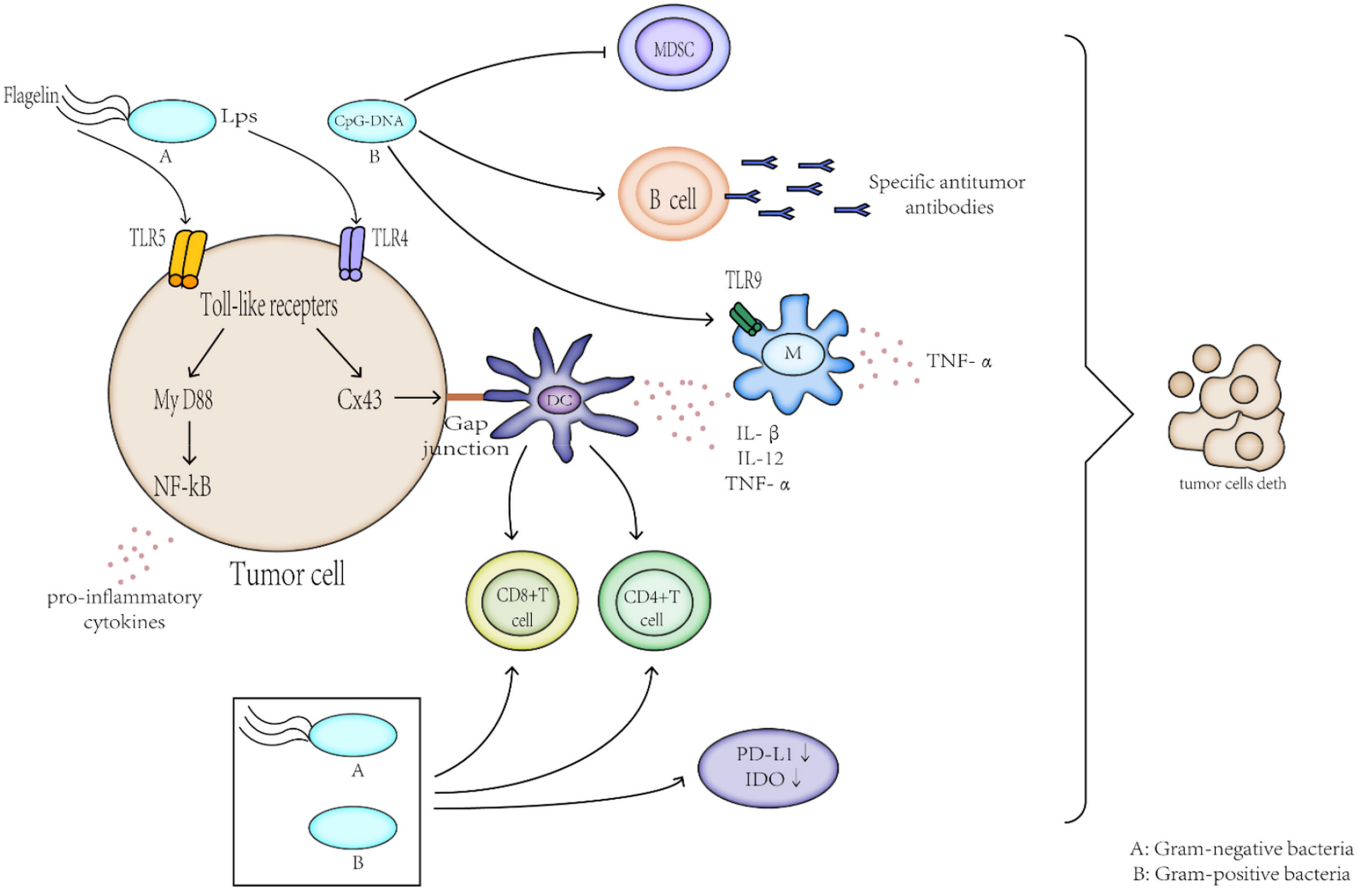
Bacteria have been shown to inhibit tumor growth through different mechanisms. Bacteria localize to the tumor microenvironment. Interactions between bacteria, cancer cells, and the surrounding microenvironment lead to various changes in tumor‐infiltrating immune cells, cytokines, and chemokines that further promote tumor regression. Different bacteria participate in the immune system in different ways, due to their different structures, they activate specific toll‐like receptor (TLR) family members to elicit distinct innate immune‐signaling cascades that ultimately translate to a comprehensive immune signature unique to each bacterial organism

Bacteria can act as an anti‐tumor agent targeting the tumor microenvironment, followed by expanding and inhibiting tumor growth. The obligate anaerobic bacteria *Clostridium* and *Bifidobacterium* can specifically target the hypoxic areas of the solid tumor harnessing the trait of requiring an anaerobic environment for survival. In this way, these bacteria can selectively colonize and reproduce, and destroy the hypoxic zone in this process.^9^ The facultative anaerobes, such as *Salmonella, and Listeria* accumulate in the tumors employing the mechanism, which follows: (1) The bacteria trapped within the messy vascular system of the tumor^10^ induce inflammation, and passively flood into the tumor. For example, studies have demonstrated that TNF‐α acts like a vascular disrupting agent in the initial stage of tumor colonization by *Salmonella typhimurium*, allowing the bacteria to be flushed into the tumor along with the blood.^11^ (2) The bacteria tend to flow into the tumor in response to the compounds produced by tumors. The motile *S. typhimurium* is easily attracted to compounds produced by the quiescent cancer cells and induces apoptosis.^12^ (3) The tumor microenvironment imparts enormous immunosuppression, which prevents the trapped bacteria from being cleared by the host immune system,^13^, ^14^ In Figure 1, the bacteria‐infected tumors inhibit tumor growth via different mechanisms.

Tumor resistance to radiotherapy and chemotherapy predominantly occurs due to the presence of the hypoxic and necrotic zones. Earlier studies have shown that radiotherapy's effectiveness depends on the partial pressure of oxygen in the tissue, as evident in the case of hypoxic cells, where the resistance to ionizing radiation is significantly higher than that of the normal cells.^15^ Therefore, the efficacy of radiotherapy is dampened by the presence of hypoxic areas within the tumor. In the case of bacterial immunotherapy, these bacteria do not affect the inherent sensitivity of the tumor cells to radiation, hence, they serve as radio‐enhancers.^16^ Combining radiotherapy with bacterial treatment might lessen the required dosage for conventional radiotherapy, therefore this combinatorial treatment can serve as a new strategy for treating malignant tumors.^16^

## 3 ACTIVATION OF THE INFLAMMASOMES

The inflammasome is a large multi‐molecular complex activating and controlling the proteolytic enzyme caspase‐1. The activation of the inflammasome is critical for the caspase‐1 dependent pro‐inflammatory cytokines and promotes its maturation. On the other hand, inflammasomes respond to pathogens and endogenous danger signals.^17^ After inflammasome activation, the inflammatory cytokines and the natural immune system are activated by the cellular signaling cascades, and in turn, cross‐activates the adaptive immune system to finally establish an antigen‐specific immune response.^18^ In bacteria‐mediated cancer therapy, the inflammasomes are activated by either of these mechanisms: Either the bacteria directly activate the inflammasomes, or the inflammasome activation occurs via endogenous danger signals released by the infected, damaged tumor cells or the phagocytosis of the damaged tumor cells.^18^, ^19^

### 3.1 The bacteria directly activate the inflammasome pathway

The bacterial outer membrane possesses structures like the lipopolysaccharide (LPS), or flagellin in gram‐negative bacteria, and lipoprotein in gram‐positive bacteria. These structures are a pathogen‐associated molecular model (PAMP) activating the toll‐like receptors (TLRs) through the surface‐bound pattern recognition receptors (PRRs), a large number of cytokines and chemokines can enter tumor tissues, by triggering a series of cell signals, which can inhibit the tumor growth.^20^ The TLR signaling pathway activation causes the increase in certain genes encoding type I interferons, different pro‐inflammatory cytokines such as TNF‐α, IL‐1, and IL‐6, as well as the anti‐inflammatory molecules, cyclooxygenase‐2 (COX‐ 2).^21^, ^22^ Besides, TLR activation induces the expression of the costimulatory molecules necessary for activating the naive T cells, which form complexes with the MHC molecules.^23^ TLRs play an important role in recognizing the self‐and non‐self‐antigens, DC maturation, and initiating the antigen‐specific adaptive immune responses.^24^ It bridges innate and acquired immunity and also plays an important role in immunotherapy and vaccination. Each category of TLR can uniquely identify the different PAMPs.^25^ The type and levels of the induced cytokines mainly depend on the type of cells activated by the TLR.

#### 3.1.1 The interaction between the LPS and TLR

LPS is a well‐known important cell wall component in gram‐negative bacteria, called endotoxin, and it is also a potent stimulator of the immune system.^26^ The TLR4 usually recognizes it; the exclusive TLR induces two different signal pathways: the MyD88‐dependent signal pathway and the TRIF‐dependent or MyD88‐independent signal pathways.^27^ Studies have shown the activation of these two pathways by TLR to be critical for maximizing the immunostimulatory potential of DCs.^28^ The TLR4 expressing macrophages and dendritic cells and other antigen‐presenting cells (APCs) get stimulated by LPS producing a large amount of pro‐inflammatory substances, cytokines, chemokines, and their receptors through the MyD88‐dependent and independent pathways.^29^ Among them, IL‐1β and TNF‐α essentially mediate the local and systemic inflammatory responses. Also, TNF‐α can induce the maturation and migration of the DCs, leading to the proliferation of the helper T cell 1 (Th1) cell lineages. Therefore, TLR4 plays an important role in cancer treatment.^27^ The specific signaling pathways involved in bacteria will be described in detail later in this article.

A study showed that purified *Salmonella* LPS can activate the TLR4 to release the IFN‐γ‐based cytokines, and increase the specific CD8+ cells, thereby, inducing tumor necrosis and enhancing specific adaptive immune responses.^30^

#### 3.1.2 The interaction between the bacterial flagella and TLR

The flagella of the gram‐negative bacteria have conserved and stable N‐terminal and C‐terminal domains (D1 and D2 domains). The D1 and D2 domains are very important for recognizing TLR5 and inducing pro‐inflammatory responses.^31^ The TLR5 exists on a variety of cells like the monocytes, macrophages, neutrophils, lymphocytes, NK cells, dendritic cells (DC), epithelial cells, and lymph node (LN) stromal cells, particularly in the DC cells of the lamina propria.^31^ Flagellin activates the cells expressing TLR5 via the MyD88‐dependent or ‐independent pathways. The interaction between flagellin and TLR5 stimulates the production of a variety of substances, including pro‐inflammatory cytokines, nitric oxide (NO), H_2_O_2_, chemokines, and host defense proteins.^32^ Flagellin and TLR5 interaction lead to the activation of some immune and non‐immune cells, including the T, B, DC, NK, and non‐lymphoid cells (like the macrophages, epithelial cells, fibroblasts, stromal cells, and neutrophils).^33^, ^34^, ^35^ This activation cumulatively enhances the immune response by producing more effective antibodies and Th1 responses. Flagellin interacts with highly immunogenic tumors to induce the Th1 response and inhibit Treg, thus, inhibiting the growth of the tumor.^31^ Flagellin is an effective immunogen capable of activating the innate immune system through the TLR5 and Naip5/6 and producing a strong T‐cell and B‐cell response.^34^ Flagellin stimulates the adaptive immune response and recruits the innate immune cells to the site of infection or immunity. It activates the T cells in two ways: directly activating the T cells or presenting the homologous antigens through the antigen‐presenting cells.^36^ Thus, to summarize, flagellin is a powerful immune activator, which can be used as an adjuvant in anti‐tumor therapy such as vaccines, with the immense prospect of research and development potential as a bacteria‐mediated anti‐tumor therapy.

#### 3.1.3 Interaction between the bacterial CpG‐DNA and TLR

The toll‐like receptor 9 (TLR9) constitutes an important pathogen recognition receptor capable of detecting and binding the bacterial DNA.^37^ TLR9 interacts with the bacterial DNA to promote immune regulation in the host. The bacterial DNA is enriched in the unmethylated CpG oligonucleotides (ODN) inducing the differentiation and proliferation of the different types of cells (including endothelial cells, monocytes, neutrophils, dendritic cells, and macrophages), regulating the Th1‐related cytokines production, activates the TLR9‐dependent complement, and triggers the innate immune responses.^38^, ^39^ According to reports, CpG‐DNA affects complement activation and regulates the immune system by upregulating the CD3, CD40, and CD83 and inducing cytokines (IL‐6 and TNF‐α), throughout the blood circulation system.^40^, ^41^ The DNA of the lactic acid bacteria promotes the TH1 response, promoting stimulation of the immune system probably due to the presence of unmethylated CpG motifs in the bacterial DNA, which are known to trigger TH1‐type immune responses by activating the toll‐like receptor 9.^42^ Studies have shown that the *Bifidobacterium* genome contains numerous conserved CpG motifs with immunostimulatory activity, but the distribution of these motifs varies in the inter‐and intra‐specific manner.^43^ Stimulating the macrophages with the DNA from *Bifidobacterium* produces numerous cytokines related to the typical TLR9 signal pathway, such as the high levels of TNF‐β, and secretes the monocyte chemoattractant protein‐1 (MCP‐1).^44^ Therefore, the high frequency of CpG motifs in the bacterial DNA might determine the immunostimulatory properties of the probiotic *Bifidobacterium*.^43^ Additionally, the CpG‐ODN can induce rapid hemorrhagic necrosis in the treated tumors. Releasing the pro‐inflammatory cytokines like the tumor necrosis factor can further induce an anticancer adaptive immune response, which ultimately eliminates most of the tumors.^45^ Furthermore, the CpG‐DNA triggers the B‐cell proliferation and differentiation to produce the T cell‐independent polyclonal antibodies.^46^ The TLR9‐dependent bacterial CpG‐DNA can induce autophagy in tumor cells both in vivo and in vitro, thereby acting as an anticancer agent.^47^ Studies have shown CpG‐ODN to activate the TLR9‐JNK/P38 signaling promoting phagocytosis and autophagy of the macrophages upon stimulation by the bacteria.^48^ The cell surface lectin, CD205 acts as a CpG ODN receptor on the dendritic and B cells, delivering the bacterial DNA to the intracellular TLR9 to participate in the specific signaling pathways.^49^ CD205 also contains a CTLD involved in the surface binding and absorption of the CpG ODN and bacterial DNA.^50^ The CD93 lectin receptor act as a receptor for the DNA or CpG ODN on the plasma membrane. It allows the bacterial DNA to be delivered to the body for promoting the signal transduction with TLR9, and enhances the recognition of LPS by TLR4, thereby, enhancing the inflammatory response of the monocytes.^51^ Therefore, they are important lectins and co‐receptors that can transfer bacterial DNA to TLR9.

### 3.2 Bacteria indirectly activate the inflammasome pathway

The damage caused by bacterial infection can damage the tumor cells and release adenosine triphosphate (ATP). The extracellular adenosine triphosphate (ATP) is a key risk‐related molecular model (DAMP) molecule, released by the damaged parenchymal cells to the extracellular mediators during inflammation.^52^ ATP directly acts on the P2X7 receptor, activating the NLRP3 inflammasome in the macrophages, and further increasing the content of the inflammatory cytokines IL‐1β, IL‐18, and TNF‐α, ultimately leading to tumor regression.^20^

## 4 ANTICANCER EFFECTS OF THE BACTERIAL METABOLITES

Some of the bacterial metabolites also have anti‐tumor effects, such as the toxin (CPE) released by the gram‐positive anaerobe *Clostridium perfringens* type A strain. Claudins (Claudins, Cldns) constitute the receptors of CPE and are the main transmembrane protein associated with epithelial malignancies. CPE binds to the highly expressed cells in its receptor and is used for eliminating the tumor cells.^8^ Therefore, CPE targeting the Cldns may induce the death of the cancer cells. A previous study has using modified CPE to treat thyroid cancer and lung cancer showed high expression of Cldns on the cell surface in both cancers. This indicated that the activity of the cancer cells was significantly reduced in vitro, and the tumor growth was inhibited in vivo, eventually, leading to tumor necrosis.^53^ Besides, a study using *Bifidobacterium longum*, secreting CPE, was found to impose a significant inhibitory effect on mice with breast cancer.^54^ Therefore, in the future, CPE can exert its potential anti‐cancer ability for cancer cells with high expression of Cldns. The gram‐negative bacteria can secrete extracellular membrane vesicles (OMV), which have the immunostimulatory ability to effectively induce, an immune response to inhibit the growth of the tumor. This effect depends on the persistence of the interferon‐γ.^55^ In another study, OMV can cause mitochondrial dysfunction, as detected by the macrophages, and can then activate endogenous cell apoptosis and inflammation.^56^ Therefore, OMV can be used as a new type of cancer immunotherapy, providing powerful and long‐lasting therapeutic effects without affecting the normal tissue cells.

## 5 BACTERIA‐ACTIVATED IMMUNE CELL POPULATION

Bacterial infection can activate complex immune cell populations in the tumor microenvironment, critically contributing to tumor regression. The immune response activated by the bacteria‐mediated anti‐tumor therapy includes innate immunity and adaptive immunity. These two types of the immune system work together to enhance and amplify the immune effect, thereby promoting tumor regression. This section has described more about some of the connections bacteria have with the immune cells (however, this is not the only section detailing the potential effects of bacteria on the immune system).

### 5.1 The roles of the innate immune cells in the bacteria‐mediated immunotherapy

Once the bacteria colonize the tumor site, it leads to immediate initiation of the natural immune response against the pathogen via the neutrophils, natural killer cells (NK), macrophages, and dendritic cells (DC). The dendritic cells (DC) are the strongest antigen‐presenting cells. Not only do they directly ingest the antigen materials and present them to the CD8 T cells to activate them. For example, *Bifidobacterium* can directly induce DC maturation and cytokine production.^57^ They also obtain the antigenic substances from the neighboring cells through gap junctions. Some studies have reported the accumulation of *Salmonella* in the tumor tissues upregulating connexin 43 (Cx43). Cx43 is the chief component of the gap junctions. The use of silent Cx43 cells showed no anti‐tumor effects pointing out that *Salmonella* induces the gap junction formation by upregulating Cx43, promoting the antigen presentation ability of the DC cells. This can induce the cytotoxic T cells to inhibit tumor growth, and prevent tumor metastasis, as well as increase the sensitivity of drug therapy and improve the treatment rate.^58^, ^59^ In addition, bacteria can induce tumor cytotoxicity to produce cell debris, which initiates tumor‐specific responses after being captured by the antigen‐presenting cells.^60^

In the early stages of *Salmonella* infection, the NK cells produce interferon‐γ, which further promotes the aggregation, activation, and cytotoxicity of the NK cells clearing the metastatic cancer cells and achieving anti‐metastatic effects.^61^

Experiments have shown that after entering the tumor tissues, *Listeria* inhibits the production of MDSC, and converts some of the MDSC subgroups infected by *Listeria* into IL‐12 producing immunostimulatory phenotypes, and enhancing the T and NK cell responses.^62^

The accumulation of bacteria in the tumor site is triggered by a strong inflammatory response, recruiting a large number of innate and adaptive immune system cells. Like *Salmonella* and *Listeria* spp, infections by *Clostridium* can also recruit the granulocytes and cytotoxic lymphocytes to the TMEs. Such recruitment leads to a significant increase in the levels of the various cytokines and chemokines at the infected sites, which can further promote the elimination of tumors.^63^

### 5.2 The roles of the adaptive immune cells in the bacteria‐mediated immunotherapy

Bacterial infection can enhance the response of the T lymphocytes, which are the most important cells involved in the anti‐tumor immune response. The hosts usually have a normal immune system. Previous studies have shown that *Salmonella enterica* serovar Choleraesuis (*Salmonella choleraesuis*) can significantly inhibit tumor growth, but in CD4+ or CD8+ T lymphocyte depleted mouse models, the inhibition of tumor growth is significantly reduced.^64^ In addition, the gut microbiota can also strongly induce the production of interferon‐γ‐producing CD8 T cells.^65^ Bifidobacterium is an active regulator of anti‐tumor immunity. Mice treated with bifidobacteria showed significant tumor suppression, accompanied by an increase in the tumor peripheral specific T‐cell infiltration and an increase in the antigen‐specific CD8+ T cells in the tumor. However, bifidobacterial treatment has no inhibitory effect on the CD8‐depleted mice, again demonstrating that its mechanism of action is indirectly achieved through the host's anti‐tumor T‐cell response.^66^ The oral bacteria killed by heating nullifies the therapeutic effect on the tumor growth and reduces the tumor‐specific T‐cell infiltration. This indicates that the anti‐tumor effect of bifidobacterial requires live bacteria.^22^, ^57^ Various bacteria rely on the different T cells to fight tumors. In a study where the mice were infected with *Escherichia coli (E. coli)* to eliminate tumors, the depleted lymphocytes verified the CD8(+) T cells as the only effector for eliminating the tumor during the induction of the tumor induction phase. In the memory stage, the CD8(+) and CD4(+) T cells work together with the tumor cells.^67^ Also, studies have shown that BCG is mainly dependent on the specific CD4 T cells, and inducing and promoting tumor elimination by increasing the interferon‐γ signaling. Therefore, the anti‐tumor effect does not require the MHC‐II restriction but requires the tumor cells to express the interferon‐γ receptors.^68^ Bacterial anti‐tumor therapy not only recruits the immune cells to fight tumors but also inhibits tumor growth by downregulating the expression of the related cells or proteins promoting tumor growth. Experiments have confirmed that the tumor size in the typhus‐infected mice regresses related to the downregulation of CD44^high^ and CD4 + CD25 + Treg cells.^69^ The non‐kinase transmembrane glycoprotein, CD44 is overexpressed in several cell types including the cancer stem cells, and is often implicated in cancer development and progression.^70^ Therefore, the targeted removal or inactivation of CD44 and Treg cells in the animal models might improve the immune surveillance in the tumor and enhance anti‐tumor immunity.

## 6 THE ROLE OF BACTERIA ON THE IMMUNE CHECKPOINT MOLECULES

The programmed death‐ligand 1 (PD‐L1) is an important immune checkpoint molecule involved in escaping the immune system in tumors. It can inhibit T‐cell proliferation, cytokine production, and cytotoxicity. PD‐L1 is reportedly expressed in many tumor tissues.^71^ Although the tumor cells express the immunosuppressive signal proteins to achieve immune escape, some bacteria have immunomodulatory capabilities against the tumors. Studies have shown that *Salmonella* can downregulate the expression of PD‐L1 in the tumor cells and inhibit tumor growth, which is related to the levels of the phosphorylated protein kinase B (p‐AKT), phosphorylated mammalian target of rapamycin (p‐mTOR), and phosphorylated p70 ribosomal S6 kinase (p‐p70S6K).^72^ In another study, when *Salmonella* was co‐treated with interferon‐γ, it reduced cytokine production by increasing the expression of PD‐L1 in the intestinal epithelial cells.^73^ *Salmonella* downregulates indoleamine 2, 3‐dioxygenase 1 (IDO), another immune checkpoint factor, resulting in decreased kynurenine synthesis and increased CD8+ T‐cell infiltration.^74^ When *Salmonella* is treated with the inhibitory molecule indoleamine 2,3‐dioxygenase, the efficacy of the immune checkpoint blockade (ICB) can be improved.^75^ In addition, studies have reported that when bifidobacterial was orally administered alone, it demonstrated a therapeutic effect similar to that evident upon using the PD‐L1‐specific antibodies. On the other hand, Bifidobacterium combines with the anti‐PD‐L1 antibody and induces the aggregation of the CD8+ T cells slowing down the growth of melanoma. This might be related to the enhanced function of the dendritic cells by bacteria, which leads to the enhanced infiltration of the CD8 + T cell and accumulation in the tumor microenvironment.^57^ These observations suggest that the symbiotic microbiome might influence the anti‐tumor immunity of human cancer patients. Interactions between the host and microbial factors can lead to subtle differences between human health and disease. The human gut microbiota contains about 3 × 10^13^ bacteria, most of which are commensal.^76^ The gut microbiota composition has emerged as a major factor with profound effects on the peripheral immune system, including that in cancer.^77^ The effects of the gut microbiota on the tumor immune responses are mainly manifested in the activation of regulatory T‐cell proliferation and differentiation; antimicrobial peptide expression; induction of IgA expression, regulation of systemic inflammation, and impact on microbial metabolism and bacterial translocation.^78^, ^79^ The host‐gut microbial symbiosis has important effects on the local and remote immune systems, significantly affecting the efficacy of immunotherapy in cancer patients. The immune checkpoint antibodies are less effective in cancer immunotherapy with antibiotic use, whereas better efficacy can be observed in the presence of specific gut microbes. Several independent retrospective analyses in populations of patients with metastatic lung, bladder, and kidney cancer have shown the adverse effects of different classes of antibiotics taken before and after PD‐1/PD‐L1 therapy on immunotherapy.^80^ To corroborate these experimental results, the fecal samples from the patients were transferred into sterile or antibiotic‐treated SPF mice, followed by inoculation of the mice with syngeneic tumors, followed by treatment with CTLA‐4 and/or PD‐1/PD‐L1 mAbs, which would result in improving the efficacy of immunotherapy.^80^, ^81^, ^82^, ^83^ Several completed (e.g., Baruch EN et al., Science, 2021) and ongoing (NCT03341143) trials evaluated the role of fecal microbiota transplantation in combination with the immune checkpoint inhibitors, suggesting that the gut microbiota may be the ICB modulators of the therapeutic response.^84^, ^85^ Additionally, an ongoing trial (NCT03829111) shows that a live bifidobacterial product (CBM588) to improve the clinical outcomes in patients with metastatic renal cell carcinoma treated with nivolumab–ipilimumab, further hinting that the gut microbiome might influence the cancer patients' response to the immune checkpoint inhibitors.^86^ However, there is limited consensus on the specific microbiome signatures associated with the clinical benefit of the immune checkpoint inhibitors. A cohort study of patients with melanoma showed that the bacteria associated with a favorable response were limited to the Actinobacteria phylum and the Lachnospiraceae/Ruminococcaceae families of Firmicutes. In contrast, the gram‐negative bacteria were associated with some adverse outcomes.^87^ Other investigators have found the gut microbiome to be associated with the response to immune checkpoint inhibitors, but it is cohort‐dependent.^88^ In short, the role of the human gut microbiota in the response to the immune checkpoint inhibitors is extremely complex, beyond the simple presence or absence of distinct microbial species in the responders and non‐responders. The complex interplay between the gut microbiota and cancer immunotherapy response should be further explored in the future. Therefore, manipulation of the bacterial populations combined with the use of immune checkpoint inhibitors might serve as another immunotherapy for treating cancer.^89^

## 7 IMPORTANT SIGNALING PATHWAYS INVOLVED IN THE ANTI‐TUMOR BACTERIA

The bacteria‐mediated tumor therapy changes the signaling pathways. Certain gram‐negative bacteria (such as *Salmonella*) can transfer the flagellin to the cytoplasm of the host cells through the type III secretion system. In this way, they can control the different host cell signal transduction pathways.^90^ Delivery of the flagellin in the host cytoplasm was first recognized by NAIP (NLR family, apoptosis inhibitor protein) family proteins NAIP5 and NAIP6. Subsequently, the NAIP5/6 complex bound to the flagellin interacts with the NOD‐like receptor NLRC4, and results in the caspase‐1 activation, cleaving the pro‐interleukin 1β into active IL‐1β.^91^, ^92^ These pro‐inflammatory cytokines are therefore very important for infection and damage in the defense response of the host. The phosphatidylinositol 3‐kinase (PI3K)/AKT/mammalian target of the rapamycin (mTOR) signaling pathway is critical for regulating cell survival, growth, and proliferation.^93^ Studies have confirmed that *Salmonella* induces regression of the tumor cell by downregulating the AKT/mTOR pathway, which induces the autophagy signaling pathway and eventually leads to cell death.^94^ Bacteria can induce the innate immune response through the TRL‐MyD88 signaling pathway or the MyD88‐independent pathway. The MyD88‐dependent pathway activates the downstream adaptor molecules of the MAP and IkB kinase pathways. It then induces the transcription factors‐ AP‐1 and NF‐κB and subsequently activates a variety of genes essential for the host defense.^95^ The MyD88‐independent pathway involves the TLR5/TLR4 heterodimer complex, activating the cells through the TRIF‐mediated pathway, rather than forming the MyD88 adapter molecules. The TRIF activation induces the antiviral cytokine IFN‐β production through the transcription factor of IRF3. Subsequently, IFN‐β activates the STAT1 transcription factor, thereby promoting the transcription of the inducible nitric oxide synthase (iNOS) gene and producing nitric oxide.^96^ The adaptor protein, MyD88 regulates the signal transduction of IL‐1R and IL‐18R, the main downstream mediators of the TLR5 and Naip5/6 Nlrc4‐inflammasome.^34^ Angiogenesis is critical for the metastasis of most tumors. *Salmonella* inhibits tumor angiogenesis by downregulating the vascular endothelial growth factors mainly through the phosphorylated protein kinase B (p‐AKT)/phosphorylated mammalian target of the rapamycin (mTOR) pathway. This reduces the HIF‐1α and downregulates its upstream signal‐mediated protein kinase B (AKT) thereby preventing angiogenesis and inhibiting tumor growth.^97^ Gut microbiota, especially bifidobacteria, preferentially colonize the tumor sites and increase the cross‐reactivity of the dendritic cells for promoting immunotherapy by a stimulator of interferon genes (STING) and interferon‐dependent fashion.^22^ STING is a cytoplasmic receptor that can sense exogenous and endogenous cytoplasmic cyclic dinucleotides (CDN) at the same time, and activate the interferon regulatory factor 3 (TBK1/IRF3), NF‐κB, and signal transducer and activator of transcription 6(STAT6)signaling pathway induce type I interferon and proinflammatory cytokine response.^98^ The STING pathway connects the cytoplasmic nucleic acid with the transcription reaction, leading to the production of the type I interferon independent of MyD88, and this pathway can also be associated with the enhanced activation of the dendritic cells and tumor antigen‐specific CD8^+^ T cells.^98^

## 8 CONCLUSIONS AND FUTURE PERSPECTIVES

Most solid tumors have hypoxic areas, and hypoxia predominately accounts for the failure and ineffectiveness of radiotherapy and chemotherapeutic treatments. In recent years, an increasing number of studies have focused on bacteria due to their properties in preventing tumor growth. The use of bacteria, as a modality has been promoted for treating cancer, based on the evidence that they selectively target and proliferate in the hypoxic area of the tumor. Moreover, bacteria are also pathogenic to the tumor cells but induce negligible toxicity to the normal cells. Moreover, the bacterial anticancer agents have proven cytotoxic activity against multidrug‐resistant cancer cells. Although bacteria have many advantages in treating cancer, there are fraught with caveats, like the inherent pathogenicity, DNA instability, and short half‐life. This makes the clinical application of bacterial anti‐tumor therapy a challenging feat.^99^ Nevertheless, bacteria‐mediated tumor therapy is endowed with great potential and hence has attracted the attention of researchers worldwide in recent years. Currently, genetic engineering is being used to modify bacteria concerning cell toxicity, half‐life, and stability. In addition, some bacteria can inhibit tumor growth, without any direct cytotoxic effect on the tumor cells. Therefore, such bacteria can be used as ideal carriers for anti‐cancer drug delivery and studies are in this promising direction are still under development. Here, we have listed the anti‐tumor activities of the bacteria that are used as the carriers for delivering anti‐cancer drugs as shown in Table 1.

**TABLE 1:** Anti‐tumor activity of the bacteria as carriers

Bacteria	Tumor model	Anti‐cancer mechanism	Ref:
*Salmonella typhimurium* (SL7207)	Melanoma (B16‐F10)	As a vaccine carrier, it activates the CD4+ and CD8+ T‐cell responses increase the production of specific Th1 cytokines, increases the tetramer cells, and inhibits lung metastasis	^(^ ^100^ ^)^
*Salmonella typhimurium* (VNP20009)	Murine T‐ALL cell line L1210	Increase the levels of TNF‐α, IFN‐γ, and CXCL‐10, and upregulate NKs, CD4+ Th1 type cells, and CD8+ T cells that produce IFN‐γ	^(^ ^101^ ^)^
*Salmonella typhimurium* (A1‐R)	Lung cancer (Lewis), Melanoma (B16), Pancreatic cancer	Destroy the tumor blood vessels. Promotes the CD8 + T‐cell infiltration and inhibits tumor growth and metastasis	^(^ 102, 103 ^)^
Listeria monocytogenes (Lmat‐LLO and Lm[ct])	A genetically engineered mouse model (GEMM) of melanoma	Increasing the level of intracellular Ca2 + , produces high levels of ROS, enhances specific CTL responses, and decreases tumor growth	^(^ ^104^ ^)^
Listeria monocytogenes (Lm ‐LLO‐Flk‐1)	Breast cancer (NT‐2 and J774A.1 cells)	A fetal liver kinase 1 (Flk‐1), as fusion proteins with the microbial adjuvant, LLO. Targeting the endothelial cells by Flk‐1 can induce the epitopes to spread to the endogenous tumor proteins, reduce the tumor micro‐vessel density, and cause tumor death	^(^ ^105^ ^)^
Bifidobacterium (displaying WT1 protein)	Leukemia (C1498‐WT1)	Significantly induce the tumor cells to infiltrate the CD4+ T cells and CD8+ T cells, producing systemic WT1‐specific cytokines, and cytotoxic activity mediated by the WT1 epitope‐specific cytotoxic T‐lymphocytes, and significantly inhibiting the WT1 gene expression for tumor growth in mice	^(^ ^106^ ^)^
E. coli Nissle 1917	colorectal cancer (CT26)B‐cell lymphoma (A20)	Checkpoint inhibitors delivered with probiotics can activate T cells, produce a remote effect, and increase the memory population of T cells throughout the body	^(^ ^86^ ^)^

However, most of the studies on bacteria‐mediated cancer treatment are still in the pre‐clinical stages and clinical trials have not yet been carried out widely. Therefore, more clinical trials on such bacteria are necessary for the future. Previous studies have already established that monotherapy is not very effective in treating cancer. Hence, combining bacteria with other treatments has the potential advantages of effectively treating tumors. Although the main mechanisms used by bacteria to treat tumors are different, it is clear that combining bacteria with the other conventional treatment can provide unique immunotherapy and can also enhance their immunity through the complex genetic engineering of the bacterial strains. Some bacteria can activate multiple TLR pathways, recruiting a large number of cytokines to reach the site of the tumor and induce specific CD4 + T and CD8+ T cells to attack the tumor. In addition, certain bacteria can downregulate the expression of the immune checkpoints. Therefore, combining bacteria with immunotherapy would produce a synergistic effect. So far, immunotherapy is considered a promising strategy for cancer therapy and bacteria are potentially one of the most effective weapons. Researchers should realize its unique advantages to redefine the existing cancer treatment into a more effective one.

## CONFLICT OF INTEREST

The authors declare that they have no potential conflict of interest.

## AUTHOR CONTRIBUTIONS

JunYing Fan was involved in conceptualization, data analyses, and writing. Yuan Huang was involved in conceptualization, writing, and reviewing. Yi Li was involved in conceptualization, data analyses, and reviewing. Tobias Achu Muluh was involved in conceptualization, reviewing, and editing. ShaoZhi Fu and JingBo Wu were involved in study concept and design, draft manuscript preparation and analysis and interpretation. All authors reviewed the results and approved the final version of the manuscript.

## ETHICS STATEMENT

This review does not contain human or animal subject, thus, informed concern in not applicable.

## Data Availability

All data that support the findings of this study are openly available in with the corresponding authors.
